# Association of sub-microscopic malaria parasite carriage with transmission intensity in north-eastern Tanzania

**DOI:** 10.1186/1475-2875-10-370

**Published:** 2011-12-16

**Authors:** Alphaxard Manjurano, Lucy Okell, Tedson Lukindo, Hugh Reyburn, Raimos Olomi, Cally Roper, Taane G Clark, Sarah Joseph, Eleanor M Riley, Chris Drakeley

**Affiliations:** 1 Faculty of Infectious and Tropical Diseases, London School of Hygiene & Tropical Medicine, WC1E 7HT, London, UK; 2 Joint Malaria Programme, Kilimanjaro Christian Medical Centre, P.O.BOX 2228, Moshi, Tanzania; 3 Department of Epidemiology and Public Health, London School of Hygiene & Tropical Medicine, WC1E 7HT, London, UK

## Abstract

**Background:**

In malaria endemic areas, individuals are frequently asymptomatic and may be undetected by conventional microscopy or newer, rapid diagnostic tests. Molecular techniques allow a more accurate assessment of this asymptomatic parasite burden, the extent of which is important for malaria control. This study examines the relative prevalence of sub-microscopic level parasite carriage and clonal complexity of infections (multiplicity of infection) over a range of endemicities in a region of north-eastern Tanzania where altitude is an established proxy of malaria transmission. The PCR prevalence was then compared against other measures of transmission intensity collected in the same area.

**Methods:**

This study used 1,121 blood samples collected from a previously conducted cross-sectional malario-metric survey during the short rainy season in 2001 from 13 villages (three at < 600 m, four at 600-1,200 m and six at > 1,200 m in altitude above sea level). Samples were analysed by PCR for carriage of parasites and multiplicity of infection. These data were compared with other measures of transmission intensity collected from the same area.

**Results:**

Parasite prevalence was 34.7% by PCR and 13.6% by microscopy; a 2.5-fold difference in line with other recent observations. This fold difference was relatively consistent at the different altitude bands despite a marked decrease in parasite prevalence with altitude: < 600 m 70.9 *vs *28.6, 600-1,200 m 35.5 *vs *9.9, > 1,200 m 15.8 *vs *5.9. The difference between parasite prevalence by PCR was 3.2 in individuals aged between 15 and 45 years (34.5 *vs *10.9) compared with 2.5 in those aged 1-5 (34.0 *vs *13.5) though this was not statistically significant. Multiplicity of infection (MOI) ranged from 1.2 to 3.7 and was positively associated with parasite prevalence assessed by both PCR and microscopy. There was no association of MOI and age.

Village level PCR parasite prevalence was strongly correlated with altitude, sero-conversion rate and predicted entomological inoculation rate.

**Conclusions:**

Asymptomatic, low density, multi-clone malaria infection was common in this study area. These infections are important as potential contributors to the infectious reservoir of parasites and need to be identified by control programmes especially in this era where malaria elimination is a focus. High throughput standardized PCR approaches are needed to identify individuals who are malaria carriers.

## Background

Microscopy is routinely used for malaria diagnosis and epidemiological studies [1,2]. However, it has limitations due to the subjective nature and sensitivity of slide reading and its time-consuming nature when carrying out studies involving a large number of individuals [1,3-5]. There is an increase in the use of rapid diagnostic tests (RDTs) that are based on detection of parasite antigens, although they too have limitations related to sensitivity and discriminating current from recent infections [2]. Molecular diagnostic tools, such as those based on PCR, have 10-100 greater sensitivity compared to microscopy and have been used increasingly for assessing infection [4-6].

In malaria endemic areas, asymptomatic malaria parasite carriers especially adults are not uncommon and, as potential gametocyte carriers, represent an important reservoir for malaria transmission [7]. Many of these asymptomatic infections are present at densities below the limit for microscopic detection and, therefore, use of microscopy is likely to lead to underestimation of the malaria burden. Indeed, in a meta-analysis of community-based studies that use PCR detection of parasites, Okell et al showed that microscopy only detected 50% of the infections identified by PCR [8].

This current study was conducted in order to examine the relationship between PCR and microscopy in a defined geographical area where altitude has been shown to be a proxy for malaria transmission intensity [9]. Risk factors for PCR carriage were examined at the individual and village level and the correlations between PCR prevalence and other measures of transmission intensity were examined.

## Methods

### Study site and sample collection

**Figure 1:**
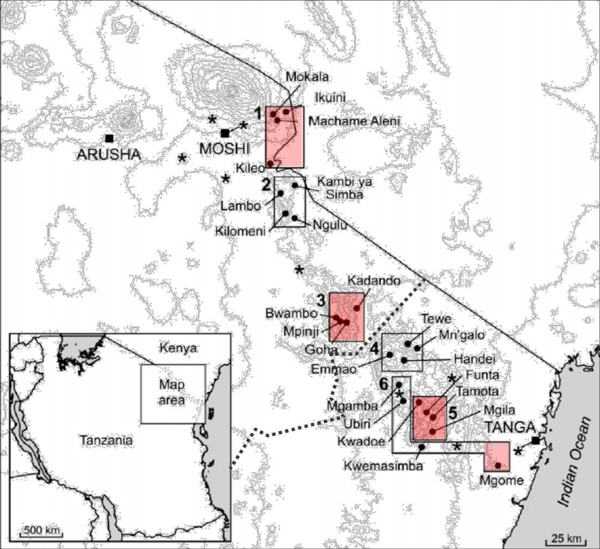
**Map of the study villages**. Each box defines a transect of four villages. The contour lines represent 400 m altitude bands. Asterisks represent locations of the nine meteorological stations, from which rainfall and temperature data were used. The dotted line indicates the regional (Kilimanjaro and Tanga) boundary. The villages in red were selected for analysis in this study. Those in the Kilimanjaro region include: Mokala, Machame Aleni, Ikuini, Kileo (Rombo transect), Bwambo, Mpinji, Lower Goha, Kadando (South Pare transect) and those in the Tanga region include: Kwadoe, Funta, Tamota, Mgila (West Usambara transect), and Mgome (Tanga coast).

The study area, study design, and sampling have been described elsewhere with parasitological, haematological, serological and entomological measures all demonstrating malaria transmission intensity decreases with altitude [9-11]. Briefly, the study area is based in north-eastern Tanzania and runs from Kilimanjaro, through the Eastern Arc Pare and Usambara Mountains to Tanga on the coastal plain. Malaria prevalence has been shown to decrease with altitude and with distance from the coast, which is linked to average annual precipitation. In 2001, cross-sectional surveys were conducted during the short rainy season (October to November 2001) and finger-prick blood samples were collected into EDTA-coated tubes from an age-stratified sample of approximately 250 people from each of 13 villages (Figure 1) from three different age groups: 0-4 (n = 80), 5-14 (n = 80) and 15-45(n = 90) years old. The villages were situated at differing altitudes and in transects selected to be as similar as possible in terms of ethnic group, socio-economic status and accessibility to health care. Full details of demographic and clinical procedures can be found in Drakeley et al [9].

For PCR analysis, samples were chosen at random from the 13 villages to include a minimum of 60 samples from each village, with 20 from each of three age groups: 0-4, 5-14 and 15-45 years old. A total of 1,121 samples were analysed for the carriage of *Plasmodium falciparum *using nested PCR parasite. Prevalence by microscopy had been determined previously and results published in Drakeley et al [9].

### DNA extraction and polymerase chain reaction

DNA was extracted from the archived, pelleted samples, using Nucleon kit protocol for DNA extraction according to manufacturer instructions http://www.tepnel.com. A nested PCR was used to amplify species-specific sequences of the small sub-unit ribosomal ribonucleic acid (18S SSU rRNA) genes of *P. falciparum *as described [12,13]. DNA from the culture of *P. falciparum *(3D7 strain) as a positive control, DNA from blood samples of an individual never exposed to malaria as a negative control, and no template as a negative control, were included in each set of PCR for quality control. The PCR was carried out in a tetrad thermo-cycler (PTC-0240, The DNA engine Tetrad2^® ^Thermal Cycler, Bio-Rad, Hercules, California, USA), followed by gel electrophoresis to assign individuals as either parasite positive or negative.

### MOI genotyping

Nested PCR was carried out to determine the numbers of *msp2 *(FC27 and IC/3D7) clones. To amplify DNA, a primer pair corresponding to the outer conserved region of the polymorphic repetitive block 3 of *msp2 *was used. Then the PCR product was re-amplified in a pair of reactions using primers specific for FC27 and IC/3D7 allelic types of *msp2 *as described by Snounou et al [14]. Number of products, corresponding to number of infecting FC27 and IC/3D7 clones, was counted after visualization on ethidium bromide (EtBr) stained 2.5 and 1.5% agarose gel, respectively.

### Data analysis

The difference in PCR-based parasite prevalence in different villages was assessed by using chi-square and logistic regression to determine the association of parasite prevalence by PCR with different predictors as independent variables, such as age, distance from the coast (a crude proxy for rainfall) and altitude. PCR prevalence and MOI were examined for correlation with altitude, sero-conversion rate (SCR) for age related acquisition of antibodies to apical membrane antigen-1(AMA-1)[15] and measured and extrapolated entomological inoculation rate (EIR) [15] that had been collected previously. Regression analysis was conducted in Stata using the 'svy' function to allow for stratified analysis with village as the primary sampling unit.

### Ethical considerations

Ethical clearance was obtained from the London School of Hygiene & Tropical Medicine (LSHTM), Kilimanjaro Christian Medical Centre, Tanzania (KCMC) and the Tanzanian National Medical Research Institute (NIMR).

## Results

### Microscopy and PCR results

The comparison between the 1,116 samples with both PCR and microscopy results is shown in Table 1. Of these, 62.8% were negative and 9.2% positive by both methods, however, PCR identified more parasite-positive individuals than microscopy: 34.4 *vs *12.0% respectively. Thirty-one of 1,116 (2.7%) samples were positive by microscopy but negative by PCR; the majority of these samples (22) had parasite densities ≤ 80 parasites/μl. The samples that were microscopy positive but *P. falciparum *negative by PCR were analysed for other species. Two of the samples were *Plasmodium malariae *positive.

**Table 1:** Comparison of microscopy and PCR results for detection of *Plasmodium falciparum *infection

	PCR		
**Microscopy**	**Negative**	**Positive**	**Total**
	
	**n**	**n**	**N**

Negative	701	281	982

Positive	31	103	134

**Total**	732	384	1,116

Using PCR as a gold-standard technique for malaria parasite detection, the sensitivity and specificity of microscopy were 26.8% (103/384) and 95.8% (701/732), respectively. The positive and negative predictive values were 78.4% (105/134) and 71.2% (699/982).

### Comparison between microscopic and PCR parasite prevalence in the study area

**Figure 2:**
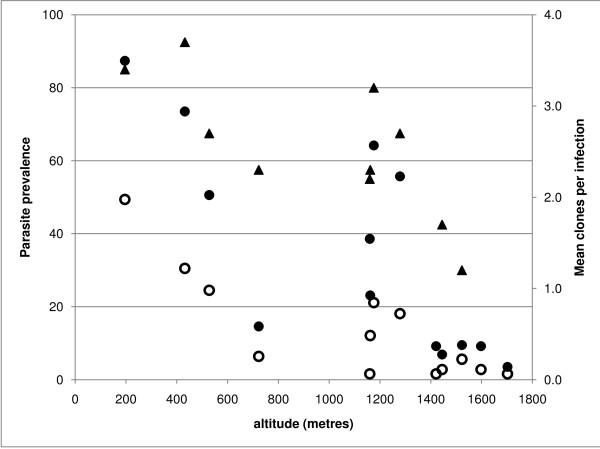
**Relationship between village level parasite rate by microscopy and PCR, multiplicity of infection and altitude Malaria prevalence by microscopy (open circles) and by PCR (closed squares) is plotted on Y1 axis**. The mean number of clones per infection (closed triangles) is plotted on Y2 axis. R2 values are 0.69, 0.59 & 0.56 for microscopy and PCR, multiplicity of infection respectively.

The 2.5-fold difference in the prevalence of parasitaemia by PCR compared to microscopy is in line with other recent observations. The difference was observed in all villages at different altitudes (Table 2 and Figure 2) and was relatively consistent at the different altitude bands despite a marked decrease in parasite prevalence with altitude: < 600 m 70.9 *vs *28.6, 600-1,200 m 35.5 *vs *9.9, > 1,200 m 15.8 *vs *5.9. The difference between parasite prevalence by PCR was 3.2 in individuals aged between 15 and 45 years (34.5 *vs *10.9) compared with 2.5 in those aged 1-5 (34.0 vs 13.5), though this was not statistically significant. The highest prevalence was observed in the middle (5-14 years) age group and was lowest in adults (15-45 years) (Table 3).

**Table 2:** Comparison of malaria prevalence by microscopy and PCR at village levels in the study area

Transect (region) Village	Mean altitude (m)	PR by microscopy, %(n/N)	PR by PCR, %(n/N)	MOI, Mean(range)	SCR*	EIR
**Rombo (Kilimanjaro**)						

Mokala	1702	1.6 (4/250)	3.5 (3/85)	-	0.031675	0.020172

Machame Aleni	1421	1.6 (4/251)	9.2 (8/87)	-	0.016603	0.100081

Ikuini	1160	1.6 (4/250)	38.6(34/88)	2.3 (1-6)	0.046994	0.445583

Kileo	723	6.4(16/250)	14.6(13/89)	3.0 (1-4)	0.1229	5.379136

**South Pare (Kilimanjaro)**						

Bwambo	1598	2.8 (7/250)	9.2 (7/76)	-	0.024609	0.0367

Mpinji	1445	2.8 (7/250)	6.9 (6/87)	1.7(1-3)	0.065053	0.087784

Goha(Lower)	1162	12.1(30/249)	23.1 (21/91)	2.2(1-4)	0.123325	0.438028

Kadando	528	24.5(61/249)	50.6 (44/87)	2.7(1-7)	0.170399	16.34673

**West Usambara (Tanga)**						

Kwadoe	1523	5.6 (14/250)	9.5(8/84)	1.2(1-2)	0.046388	0.044549

Funta	1279	18.1 (45/249)	55.7(49/88)	2.7(1-7)	0.184438	0.282417

Tamota	1176	21.1 (52/246)	64.2(52/81)	3.0(1-5)	0.103647	0.810688

Mgila	432	30.5 (74/243)	73.5(61/83)	3.2(1-6)	0.236782	39.10021

**Tanga Coast (Tanga)**						

Mgome	196	49.4(122/247)	87.4(83/95)	3.4(1-8)	0.28871	119.5014

**Total**		13.6(440/3234)	34.7(389/1121)			

*SCR *Sero-conversion rate, *EIR *Entomological inoculation rate, *MOI *Multiplicity of infection, *PR *Parasite prevalence rate.* data obtained from Corran et al [15] and Drakeley et al [9]

**Table 3:** Prevalence of malaria infection by age in each altitude band by both PCR and microscopy

Altitude band	< 600 m		600-1200 m		> 1200 m	
**Age group (years)**	**Microscopy Prevalence (%)**	**PCR Prevalence (%)**	**Microscopy Prevalence (%)**	**PCR Prevalence (%)**	**Microscopy Prevalence (%)**	**PCR Prevalence (%)**

0-5	39.0	74.5	9.5	32.5	5.2	13.7

6-10	48.0	72.0	13.7	36.1	6.2	18.8

11-15	43	65.8	10.1	34.0	2.8	14.9

16-20	35.7	100*	11.0	37.0	10.0	11.5

21-25	21.2	52.9	10.3	44.4	6.8	6.3

26-30	13.5	73.7	15.4	20.8	4.5	26.8

31-35	25.4	68.8	5.3	40.0	4.7	8.3

36-40	17.1	84.6	4.9	36.8	4.9	14.7

41-45	14.3	45.5	8.8	37.5	8.2	27.3

*only seven samples analysed

### Mixed parasite species infections

This was assessed in samples that were microscopy negative but *P. falciparum *PCR positive, and microscopy positive but PCR *P. falciparum *negative. A total of 117 samples were analysed for mixed infections by PCR, 12% of individuals had co-infections of *P. falciparum *and either *P. malariae *or *P. ovale*, and all were from lowlands. None of the samples analysed was *Plasmodium vivax*. The majority of samples (85.7%) with mixed infection were of *P. falciparum *and *P. malariae*. Six samples had *Plasmodium ovale *infection and out of these, four samples had mixed infection of *P. falciparum, P. malariae *and *P. ovale*.

### Factors related to carriage of sub-microscopic parasites in the study area

After excluding individuals who were microscopically parasite positive, a total of 982 individuals were further involved in the analysis using logistic regression. In uni-variate logistic regression (Table 4), residents in the Tanga region were five-fold more likely to carry sub-microscopic parasites (OR, 5.00 [95%CI, 3.72-6.73]; *p *< 0.0001). This was also apparent within the Tanga region with both West Usambara and Tanga coast (Mgome village) transects, which were associated with increased carriage of sub-microscopic parasites; the magnitude being approximately four-fold (OR, 4.46 [95% CI, 0.88-22.49]; *p *= 0.067) and more than ten-fold (OR, 23.86 [95% CI, 7.00-81.29]; *p *< 0.0001) respectively. Analysis based on altitude band, regardless of region, indicated that individuals living in areas where the altitude was 600 m-1,200 m (OR, 0.28 [95%CI, 0.08-1.01]; *p *= 0.052) and above 1,200 m (OR, 0.10 [95%CI, 0.02-0.43]; *p *= 0.005) were less likely to carry sub-microscopic parasites as compared to individuals living in the area of < 600 m. Use of bednets, sex, fever and treatment were not associated with sub-microscopic parasite carriage in individuals (*p *> 0.05).

**Table 4:** Uni-variate and multi-variate analysis of factors associated with sub-microscopic *Plasmodium falciparum *infection*

	Univariate analysis		Multivariate analysis	
	**OR(95%CI)**	***p*-value**	**OR(95%CI)**	***p*-value**

**Age group, years**				

0-4	1.00	-	1.00	-

5-14	0.99(0.70-1.41)	0.947	0.83 (0.50-1.58)	0.438

15-45	1.18(0.79-1.77)	0.375	1.00 (0.70-.41)	0.981

**Transect/distance from ocean**				

Rombo	1.00	-	1.00	-

South Pare	1.21 (0.23-6.36)	0.802	1.01 (0.47-2.16)	0.985

West Usambara	4.46 (0.88-22.49)	0.067	4.80 (1.79-12.86)	0.005

Tanga coast	23.86(7.00-81.29)	< 0.0001	8.61 (2.36-31.40)	0.003

**Altitude band**				

< 600 m	1.00	-	1.00	-

600-1200 m	0.28 (0.08-1.01)	0.052	0.40 (0.14-1.14)	0.081

> 1200 m	0.10 (0.02-0.43)	0.005	0.12 (0.03-0.50)	0.007

**Bed net use**	0.70 (0.25-1.96)	0.468	0.71 (0.40-1.26)	0.218

**Sex**	1.09 (0.76-1.56)	0.629	1.13 (0.60-2.12)	0.679

**Fever**	1.07 (0.55-2.07)	0.827	0.85 (0.53-1.35)	0.450

**Treatment**	0.70 (0.43-1.15)	0.143	0.77 (0.44-1.35)	0.325

*The above analysis is after excluding individuals who were microscopy parasite positive.

*OR *Odds ratio, *CI *Confidence interval

Regression analysis was conducted in Stata using the 'svy' function to allow for stratified analysis with village as the primary sampling unit

In multi-variate analysis, after adjustment for transect of residence, age, use of bed net, sex, fever and treatment, altitude band was still significantly related to carriage of the sub-microscopic *P. falciparum*, indicating that individuals living in highlands were less likely to carry sub-microscopic parasites.

Individuals with sub-microscopic parasites had significantly lower mean haemoglobin levels (11.4 *vs *12.1 g/dl respectively, F = 19.2 *p *< 0.0001), suggestive of longer term or chronic infections. However, an interaction with of other factors (such as iron deficiency anaemia, helminth infections, HIV, red blood cell polymorphisms) that are associated with decreased levels of haemoglobin cannot be ruled out. No significant difference in prevalence of sub-microscopic parasites was seen between the sexes or in those who reported fever or recent anti-malarial treatment.

### Multiplicity of infection (MOI)

The results showed that the majority of PCR positive subjects carried multiple strains; 76.2% (189/248). The MOI in PCR positive samples was significantly higher in high transmission/low altitude area (*P *= 0.025). There was no significant association between age and MOI (*p *> 0.05).

The distribution of *msp2 *allelic families with more than one clone, differed between the two allelic families; 46.8% (116/248) of parasites from 3D7/IC, 37.9% (94/248) of parasites from FC27 and 55.6% (138/248) of clones from both allelic families.

### The use of different measures to correlate malaria and transmission intensity

Comparison of the different measures of transmission intensity (Table 5, Figure 2) indicates that all measures were strongly correlated. Relevant to this study, highly significant correlation co-efficients were observed between altitude and parasite rate by PCR and by microscopy, predicted EIR and sero-conversion rate.

**Table 5:** Correlation coefficient matrix between different indicators of malaria transmission measured in each village

	Alt	PR	PCR	MOI	EIR	SCR
Alt	1.0000					

PR	-0.8328 (0.0004)	1.0000				

PCR	-0.7721 (0.0020)	0.9090 (< 0.0000)	1.0000			

MOI	-0.7481 (0.0128)	0.8111 (0.0044)	0.9156 (0.0002)	1.0000		

EIR	-0.7506 (0.0031)	0.8615 (0.0002)	0.6896 (0.0091)	0.5748 (0.0822)	1.0000	

SCR	-0.8756 (< 0.0000)	0.9333 (0.0002)	0.8607 (0.0042)	0.8136 (0.0020)	0.7718 (0.0041)	1.0000

*Alt *Altitude, *PR *Parasite prevalence by microscopy, *PCR *Parasite prevalence by PCR, *MOI *Multiplicity of infection, *EIR *Entomological inoculation rate, *SCR *Sero-conversion rate. p-values are present in brackets

## Discussion

Accurate quantification of the malaria parasite reservoir is important for control given the renewed focus on transmission reduction leading to elimination. Assessment of this reservoir by microscopy has limitations, and molecular techniques, largely based on the PCR, have been increasingly widely used to measure parasite prevalence. In this study, samples were analysed from previously conducted malario-metric surveys in an area where there is a very broad range of transmission intensity, i.e. microscopic parasite rates ranging from 2 to 50% and estimated entomological inoculation rates from < 1 to > 100 infectious bites per person per year. This variation occurs in a relatively small geographical area within similar ethnic groups, such that a number of confounders are minimized. Overall, a 2.5-fold higher parasite prevalence was observed with PCR compared to microscopy and this fold difference was consistent across the different transmission levels. The scale of the difference described here compares well with the two-fold difference documented in the meta-analysis by Okell and colleagues [8] and also from individual studies in Cambodia [16] and Solomon Islands [17].

The consistency of these observations on sub-microscopic parasitaemia suggests that this is a robust finding and one that has important implications for control measures. A study by Schneider et al [18] found that the contribution of sub-microscopic parasites to malaria transmission in individuals was similar to those individuals having microscopic gametocytes. Though gametocyte carriage was not assessed directly in this study, several studies document a high prevalence of concomitant gametocytes in individuals with asexual parasites (e.g. [19]). Although, gametocytes are likely to be present at lower densities [20] than asexual parasites, one interpretation of these observations is that using PCR for asexual parasites could be taken to equate to potential infectiousness.

Other studies [21,22] have reported that sub-microscopic carriage differs in relation to transmission intensity and age. Strong correlations with malaria transmission intensity, as characterized by altitude and transect (a proxy for rainfall which increases with increasing proximity to the Indian Ocean) [9] were observed with carriage of sub-microscopic parasites. On a micro-epidemiological level, differences were observed between villages that were similar in altitude and distance from the coast (e.g. Kadando and Kileo) showing that local factors that favour transmission will influence both microscopic and sub-microscopic parasite carriage. Similar findings have been encountered in the highlands in Kenya [23,24] indicating that small differences in altitude and other factors can influence the ecological environments of mosquitoes.

In this study, older age was not associated with increased carriage of sub-microscopic parasites. The reasons for this are not as obvious as one might expect that individuals with higher levels of immunity (i.e. older ages) would maintain parasites at lower densities [5,16]. At the lowest transmission intensity (highest altitude band) individuals older than 15 were 3.0 times more likely to have sub-microscopic parasites than younger individuals (chi sq 4.01, *p *= 0.0425) but this was not significant in multi-variant analysis. Other individual level factors such as sex, history of fever and recent treatment were not associated with parasite carriage. Use of bed nets was found to be inconsistently protective against sub-microscopic carriage; at higher transmission intensities where 50% net users were PCR positive compared to 75% of non net users at the lowest altitude, with the corresponding figures of 14 and 40% in the middle altitude band. However, the effect was not significant in stratified, multi-variate analysis.

The MOI results reported in this study provide a rough estimate of the genetic diversity of MSP2, a polymorphic marker, and suggest diversity was pronounced. Multiple parasites genotypes in individuals living in endemic areas are frequently observed, as was the case in other studies in Senegal [21,22] and Ghana [25]. A study in Ghana indicated that MOI increases the gaining of immunity and the ability to control parasitaemia and protection from subsequent clinical episodes [25]. MOI did not correlate to age; similar findings observed in a more restricted age range of 2-10 years in Senegal [22].

Sub-microscopic parasite carriage was strongly correlated with MOI and other previously collected measures of transmission intensity such as parasite prevalence by microscopy, extrapolated EIR and SCR. The relationship presented in Table 5 is for the antigen AMA-1 and, the correlation between PCR prevalence and MOI for Merozoite Surface Protein-1 is similarly strong (0.8525, *p *= 0.0004 & 0.7716, *p *= 0.017 respectively). This is not surprising as microscopy and PCR parasite prevalence were highly correlated (coefficient r^2 ^= 0.9, *p *< 0.001) and the relationship between microscopy and the other measures has been demonstrated previously [10]. However, as PCR methodology becomes increasingly more widespread, it will be important to calibrate PCR prevalence across the full range of transmission intensity. The observations made here and in other papers suggest that PCR prevalence will have a greater dynamic range than microscopy and thus will be an important parameter for assessing progress in low endemicity and pre-elimination settings.

## Conclusions

This study shows that a large number of individuals carried sub-microscopic parasites in a malaria endemic area of Tanzania. These individuals are a potential reservoir of infection in the population. There is, therefore, a need to further evaluate the burden of sub-microscopic parasites when considering elimination and eradication of malaria. Employing the multiplex PCR for the different species of *Plasmodium *and also a more sensitive method, such as *stevor *PCR suggested elsewhere [5], may be needed to resolve the discordant results of the conventional PCR and microscopy.

## Competing interests

The authors declare that they have no competing interests.

## Authors' contributions

AM, ER, and CD designed the study. HR, CD and RO were responsible for study participants recruitment, and clinical and parasitological examinations. AM, TL and SJ did the genotyping, and AM, LO, TC and CD the statistical analyses. AM, CD, TC and CR wrote the paper with major contributions of the other authors. All authors read and approved the final manuscript.
